# Effect of Haptic Training During Manual Wheelchair Propulsion on Shoulder Joint Reaction Moments

**DOI:** 10.3389/fresc.2022.827534

**Published:** 2022-04-05

**Authors:** Rachid Aissaoui, Dany Gagnon

**Affiliations:** ^1^Laboratoire de Recherche en Imagerie et Orthopédie (LIO), Centre de Recherche du Centre Hospitalier Universitaire de Montréal (CRCHUM), Montreal, QC, Canada; ^2^Département de Génie des systèmes, École de technologie supérieure (ETS), Montreal, QC, Canada; ^3^School of Rehabilitation, Université de Montréal, Montreal, QC, Canada; ^4^Pathokinesiology Laboratory (www.pathokin.ca), Institut universitaire sur la réadaptation en déficience physique de Montréal (IURDPM), Montreal, QC, Canada

**Keywords:** biofeedback, biomechanics, haptic, manual wheelchair, shoulder joint moment, mechanical efficiency

## Abstract

**Background:**

Manual wheelchair propulsion remains a very ineffective means of locomotion in terms of energy cost and mechanical efficiency, as more than half of the forces applied to the pushrim do not contribute to move the wheelchair forward. Manual wheelchair propulsion training using the haptic biofeedback has shown an increase in mechanical efficiency at the handrim level. However, no information is available about the impact of this training on the load at the shoulders. We hypothesized that increasing propulsion mechanical efficiency by 10% during propulsion would not yield clinically significant augmentation of the load sustained at the shoulders.

**Methods:**

Eighteen long-term manual wheelchair users with a spinal cord injury propelled a manual wheelchair over a wheelchair simulator offering the haptic biofeedback. Participants were asked to propel without the Haptic Biofeedback (HB) and, thereafter, they were subjected to five training blocks BL1–BL5 of 3 min in a random order with the haptic biofeedback targeting a 10% increase in force effectiveness. The training blocs such as BL1, BL2 BL3, BL4, and BL5 correspond, respectively, to a resistant moment of 5, 10, 15, 20, and 25%. Pushrim kinetics, shoulder joint moments, and forces during the propulsive cycle of wheelchair propulsion were assessed for each condition.

**Results:**

The tangential force component increases significantly by 74 and 87%, whereas value for the mechanical effective force increases by 9% between the pretraining and training blocks BL3. The haptic biofeedback resulted in a significant increase of the shoulder moments with 1–7 Nm.

**Conclusion:**

Increases in shoulder loads were found for the corresponding training blocks but even though the percentage of the increase seems high, the amplitude of the joint moment remains under the values of wheelchair propulsion found in the literature. The use of the HB simulator is considered here as a safe approach to increase mechanical effectiveness. However, the longitudinal impact of this enhancement remains unknown for the impact on the shoulder joint. Future studies will be focused on this impact in terms of shoulder risk injury during manual wheelchair propulsion.

## Introduction

Manual wheelchair (MW) propulsion is the primary mean of mobility for individuals that sustained a spinal cord injury (SCI). Although MW propulsion helps those individuals to regain certain independence and maintain or increase societal participation, it remains a very ineffective means of locomotion in terms of energy cost and mechanical efficiency (1, 2). More precisely, it has been shown that almost half of the forces applied to the pushrim do not contribute at moving the MW forward in individuals with a SCI (3–5). Earlier studies looked at the possibility of increasing the force effectiveness (i.e., the tangential component) using training methods such as visual feedback (6, 7). While de Groot et al. (6) found a significant increase in force effectiveness [mechanical effective force (MEF)] between pre- and posttraining in 10 healthy individuals, no significant augmentation was found in the study of Kotajarvi et al. (7) for 18 experienced MW users. The authors suggested that visual feedback of the average force effectiveness value might not be the optimal training strategy to improve force effectiveness during propulsion (7).

Recently, Blouin et al. (8) used an Haptic Biofeedback (HB) simulator developed by Chenier et al. (9) to increase force effectiveness in 18 experienced MW users who sustained a SCI. The authors (8) have shown a significant increase in force effectiveness using the HB. More precisely, the participants were able, on average, to increase force effectiveness by 12–15% bilaterally suggesting an interesting potential as a training tool for MW users. The HB has been previously shown to be an efficient sensory feedback tool to teach movement and force patterns in the rehabilitation of the upper limb in hemiparetic patients (10, 11). In this study, the HB is defined as the ability to our wheelchair simulator to continuously modify the direction of the force applied to the handrim during the propulsion phase in real time. In general, this operation looks as an adaptive process control. Without HB, the user propelled the wheelchair with a specific personalized pattern of propulsion as represented by his initial MEF. From that pattern, a new targeted pattern is artificially created by modifying a portion of the pattern. This forms a closed-loop control in which a resistive moment to the wheel is added or subtracted proportionally to the error signal between the targeted and the initial MEF. Since there is no visual information fed to the subject, but only proprioceptive information, i.e., a gradually resistive moment at 2 kHz, we call this control as the HB (9). In this study, the HB was modulated continuously during the propulsion phase and it takes <10 cycles when the subject senses the difference between his/her own pattern and the one imposed.

Although it seems possible to increase the MEF using an HB simulator, no information regarding the load imposed at the upper limb joints by this increase force effectiveness is yet available. Several authors suggested that propelling a MW with greater force effectiveness would yield to a greater risk exposure for the musculoskeletal structures (6, 12, 13). In the past, an effort was made to gain a better understanding of the relationship between the propulsive force effectiveness and the shoulder joint reaction moment. In fact, two simulation studies have shown that, indeed, a force close to tangential could highly increase the joint moment at the shoulder level during manual wheelchair propulsion (14, 15). More specifically, Bregman et al. (14) have shown almost a 2-fold increase in shoulder joint moments when only using the tangent force component as an input for an inverse dynamic model. However, one study (15) suggested that an improvement in the force effectiveness of around 10% would be possible without yielding significantly higher mechanical demand at the shoulder. Giving the high prevalence of secondary impairments at the shoulder among MW users, it would be interesting to determine the *in-vivo* impact of increasing force effectiveness by 10%, as suggested by Desroches et al. (15) using the HB simulator on the shoulder joint moments (16–19). We hypothesized that increasing force effectiveness by 10% during propulsion would not yield clinically significant augmentation of the load sustained at the shoulders.

## Methods

### Participants

Eighteen long-term MW users (MWUs) (16 men and 2 women) with a SCI were recruited to participate in this study (Table 1). To be included, participants had to have a complete or incomplete SCI [American Spinal Injury Association (ASIA) established a grading system called the ASIA A, B, or C] between C7 and L1 vertebral levels for 3 months or longer, use a manual wheelchair as their primary means of mobility, and be able to perform wheelchair-to-wheelchair transfers independently with or without the use of a transfer board. Participants were excluded from this study, if they had any pressure sores on the buttocks or if they reported any pain that could have hindered their propulsion biomechanics. This study was approved by the research ethics committees of the École de technologie supérieure (ÉTS) and the Center for Interdisciplinary Research in Rehabilitation of Greater Montreal (CRIR).

**Table 1:** Participants' characteristics [mean (1 SD)].

	***n* = 18**
Age	42.4
(y)	(13.9)
Height	1.73
(m)	(0.20)
Weight	77.4
(kg)	(14.1)
Time since injury	14.8
(y)	(10.1)
AIS level	1 C8, 1 T2, 2 T4, 1 T5, 2 T6, 1 T7, 1 T9, 3 T10, 1 T11, 5 T12
ASIA	13 A, 2 B, 2 C, 1 D
Gender (M/F)	16/2

### Haptic Simulator and Measurements

**Figure 1:**
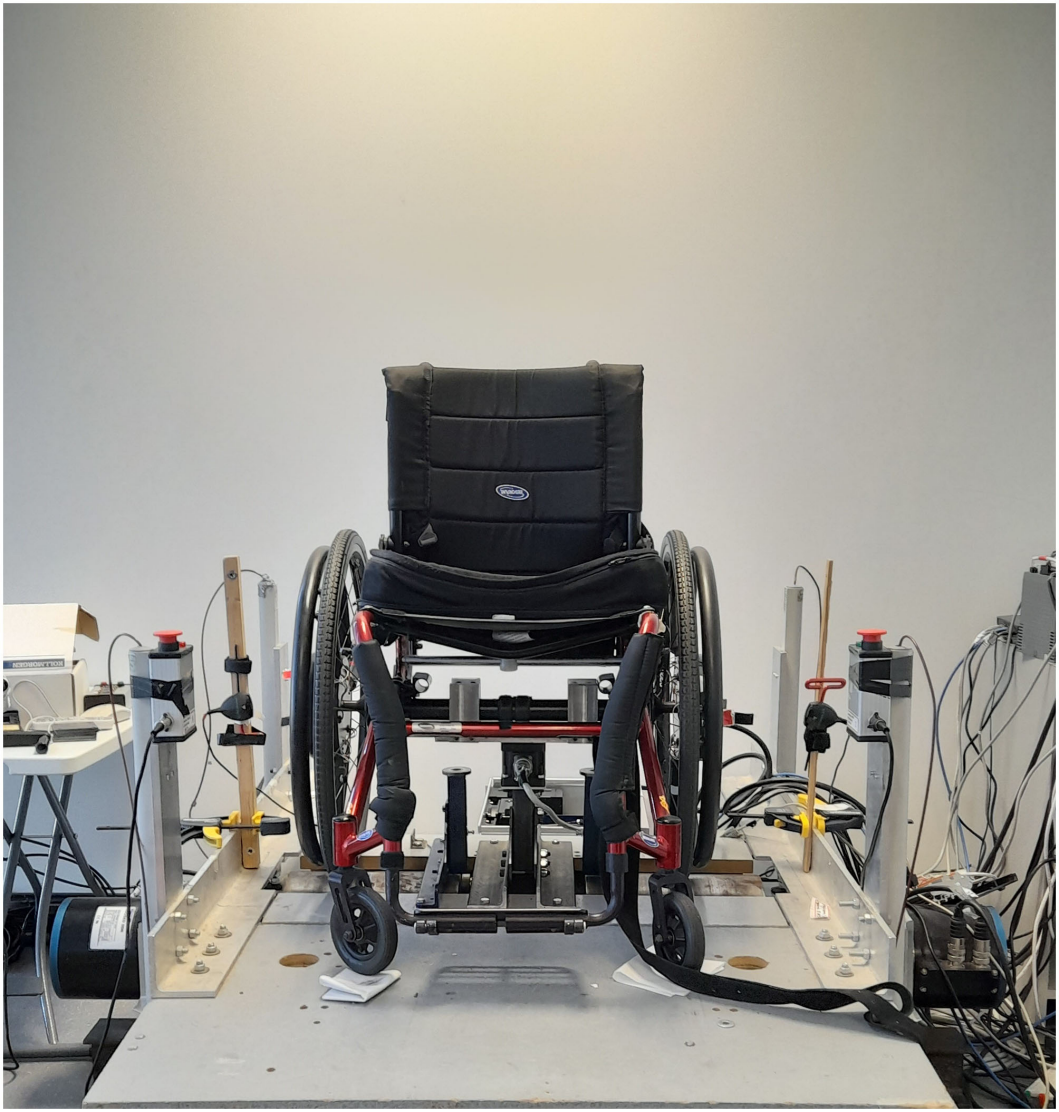
Haptic wheelchair simulator.

The experimental tasks were performed using a recently-developed haptic simulator (Figure 1) (9). Briefly, this simulator acquires real-time bilateral three-dimensional forces and moments measured with instrumented wheels (SmartWheel, Three Rivers Holding, LLC) during propulsion. The propulsive moments generated by the user about the wheel hub are used as the input for the dynamic model of a virtual wheelchair. The dynamic model, presented in a study by Chenier et al. (9), estimates the angular velocity of each rear wheel of a virtual wheelchair, which represents propulsion on a ground-level surface. Velocity controllers ensure that the angular velocities of the real wheelchair match those of the virtual wheelchair rear wheels, so that a complete haptic loop is defined between the user and the simulator. Then, based on those forces and moment as well as the desired feedback that will be described later, the haptic feedback is given to the user by two motors under each rear wheel that will induce gradual resistance during the propulsion phase when the user is not following the desired force feedback pattern (8). All the experimental tasks were performed in the *Invacare A4 Ultralight* wheelchair mounted on the simulator. Participants were assisted to transfer from their personal wheelchair to the simulation and their own seat cushion was used. The backrest angle was adjusted as close as possible to that of the participant's personal wheelchair.

The bilateral upper extremity three-dimensional kinematics was recorded with 26 reflective markers captured by six cameras VICON M460 system (Vicon Motion Systems Limited, Oxford, UK) at a sampling frequency of 120 Hz. Markers placement were similar to the one described in a study by Desroches et al. (20). The reflective markers used in this study are the ulnar and radial as well as the second and fifth metatarsal for the hand segment. For the lower arm, a cluster of 3 markers at mid-distance from the wrist and elbow joint, plus the lateral and medial epicondyles. For the humerus segment, a cluster of 3 markers at mid-distance from the elbow to the acromion markers, plus the acromion marker. For the thorax, C7 and T8 markers, plus the jugular notch as well as the sternum end.

Additionally, two reflective markers were added on the surface of each wheel to define each wheel reference systems relative to the global coordinate system. Three-dimensional forces and moments applied at the pushrim by the participants were recorded by the two SmartWheels at a sampling frequency of 240 Hz. Kinematics and kinetic data were acquired and stored on an external computer for further postprocessing.

### Haptic Biofeedback

The determination and application of the HB has been described in a previous study (8). Briefly, the HB was determined in real time and based on the difference between the actual MEF and the targeted optimized MEF (MEF_T_). MEF is the ratio between the squared tangent force component and the squared total force (2, 21). In the current experiment, the design of the curve pattern of the MEF_T_ was personalized for each subject. It corresponds to the initial MEF prerecorded pattern of the subject, which was deformed by two linear Gaussian functions in order to increase the maximal value of the original MEF by an amount of 10% (8). During the push phase of the propulsion, the participants perceive an increase of resistance as long as their actual MEF pattern deviates from the desired one MEF_T_. The intensity of the HB or the resistance felt by the participants was determined relative to their maximum voluntary propulsive moment (MVM) recorded prior to the experiment. Five relative intensities were used in the current experiment: 5, 10, 15, 20, and 25% of the participant's MVM (8).

### Pretraining

To become familiar with the haptic simulator, participants propelled freely for 1 min on the simulator. Then, two trials (named INI) without the HB were conducted at the participant's self-selected velocity for 1 min each. Kinematics and kinetic data were recorded for the last 30 s of each trial. The mean linear velocity reached during each of the two 30-s acquisition periods was calculated. If the mean linear velocity varied by more than 10% between the two trials, a third trial was recorded and trial 1 was discarded.

### Training

Training was divided into five 3-min blocks. 2-minute rest periods were included after each block. Each training blocks were corresponded to an intensity level (BL1–BL5 corresponding to 5–25% with a step of 5%, respectively) and were presented in a random order. The HB was activated 3 s after the beginning of each training block and kept active until the end of the block. Participants were told that they had to push more tangentially on the handrims to increase their MEF. Participants were also instructed to always strive for the lowest resistance possible, which indicated that their actual MEF pattern was coming closer to the target pattern MEF_T_. Kinematics and kinetics data were acquired during the last 30 s of each training trial. In addition to the HB, the average speed of each block was shown to help participants to match their velocity to the velocity achieved during pretraining.

### Posttraining

After a 2-min rest period, two posttraining trials (POST) without the HB lasting 1 min each were conducted with the same methodology used during pretraining. The only visual feedback provided was the average speed during propulsion.

### Postprocessing

For each of the experimental tasks, three-dimensional trajectories of each kinematic marker were filtered using a 4th order low-pass Butterworth filter with a cutoff frequency at 6 Hz, while pushrim forces and moments were filtered using an 8th order low-pass Butterworth filter with a cutoff frequency of 30 Hz.

### Pushrim Force Measurements

For each of the experimental tasks, the resultant force at the pushrim (Fres) and its tangential component (Ftan) were continuously calculated as well as the MEF bilaterally. The Fres was defined as the vector sum of the three force components measured by the SmartWheel. The Ftan was obtained using the point of force application method (22). The magnitude of the tangential force was estimated by dividing the moment around the medial-lateral axis of the SmartWheel by the handrim radius. The MEF was then obtained as the ratio between the tangent force component squared and the resultant force squared.

### Inverse Dynamics Software

Upper limb net joint moments and forces were estimated using an inverse dynamic method (23). The forces and moments measured by each SmartWheel, upper limb kinematics, and the mass and height of each subject are used as input for the calculation of the shoulder joint reaction forces. The segment coordinate system of the forearm and arm was defined according to the International Society of Biomechanics (ISB) recommendations (24). The segment mass, the position of center of mass, and the inertia tensor of each body segment were estimated by scaling equations based on participants' anthropometry (25). The segment length of the hand and lower and upper arms were measured based on markers. Hand segment length was defined as from the mid-distance between the ulnar and radial markers and the 2nd and 5th metatarsals. The lower arm segment was defined from the mid-distance of the elbow lateral and medial epicondyles and the mid-distance between the ulnar and radial markers. The upper arm was defined from the midepicondyle of the elbow and the center of the glenohumeral joint as defined by statistical equation from the acromion. Also, the gender and the weight of the person were used in the statistical equation in (25) to estimate the location of the center of mass of each segment as well as the moment of inertia around each axis.

The outputs of the calculation were the bilateral net joint forces and moments acting at the shoulder joints and the segment angular velocities in the global coordinate system. The net shoulder joint forces and moments represent the actions exerted by the proximal segment on the distal segment and were expressed in the joint coordinate system (JCS) proposed by (26). Positive shoulder moments were in flexion, adduction, and internal rotation, whereas positive forces were medial, anterior, and proximal.

### Data Analysis

For each trial, the 10 most repeatable push cycles were used (8, 27). The bilateral Fres, Ftan, MEF, and shoulder joint moment and force components were normalized with respect to the push phase in 101 data points and they were subsequently divided into four quartiles: Q_1_ = 0–25%, Q_2_ = 25–50%, Q_3_ = 50–75%, and Q_4_ = 75–100%. The analysis was specifically conducted on quartiles Q_2_ and Q_3_ because the HB was generally active in this portion of the push phase and also because most of the propulsion effort was provided in the middle of the push. The average of the MEF and each moment components were calculated during Q_2_ and Q_3_ for the seven experimental conditions (i.e., INI, BL1-BL5, and POST).

### Statistical Analysis

All the dependent variables (i.e., average of the Fres, Ftan, and MEF) as well as the shoulder moment components during Q_2_ and Q_3_ for the flexion/extension, adduction/abduction and internal/external rotation moments, and medial/lateral, anterior/posterior, and proximal/distal force components met the normality criteria (Shapiro–Wilk test, *p* > 0.05). Repeated measures ANOVAs were performed for the dependent variables in order to determine the effect of training intensities with a significance level set at *p* < 0.05. When a significant main effect was found, *post-hoc* analysis using dependent *t*-tests was performed between the pretraining (INI) condition and each of the five training blocks (BL1–BL5) as well as with the posttraining condition (POST). The significance level was adjusted to account for multiple comparisons using the Bonferroni corrections (*p* < 0.05/6 = 0.0083).

## Results

### Pushrim Kinetics

**Figure 2:**
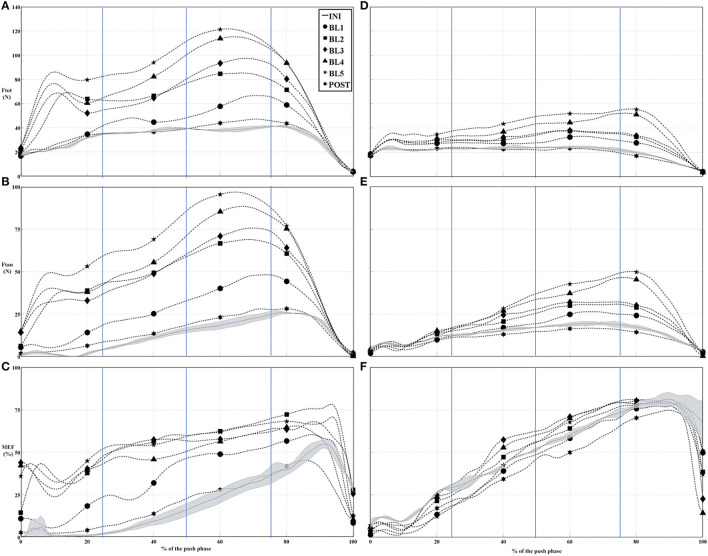
Normalized time series of the Ftot, Ftan, and mechanical effective force (MEF) for a participant that was initially inefficient **(A–C)** and a participant that had initially high MEF **(D–F)**. Vertical lines indicate each quartile separation, i.e., 25, 50, and 75%.

Table 2 shows the average of the pushrim kinetics parameters. Significant main effects were found for the Fres and Ftan at the pushrim bilaterally during both the Q_2_ and Q_3_. *Post-hoc* analysis revealed that all the force components in the INI condition were significantly lower compared to all the training blocks. Significant main effects were found for the average MEF during Q_2_ and Q_3_, as subsequent analysis revealed that the MEF in the INI condition was significantly lower compared to BL3, BL4, and BL5 (Table 2). In fact, MEF in Q_2_ varied from 33 and 35% in INI condition to 48 and 50% in BL5, respectively, for the right and left sides. During the Q_3_ interval, the MEF varied from 52 and 53% in INI condition to reach 61 and 62% in BL5 condition. We can consider here that the participants modify their MEF toward the direction of the MEF target, which corresponds to a 10% increase at the peak value of the MEF. Since the MEF has a pattern that is participant dependent, we show here that our participant learned the new imposed pattern with our simulator. Figure 2 shows the time-normalized Fres, Ftan, and MEF for a participant that had the lowest MEF and a participant that had the highest MEF at INI and their patterns for all the training blocks.

**Table 2:** Average (1 SD) bilateral resultant force at the pushrim (Fres), tangential (Ftan) force component in Newtons, and mechanical effective force (MEF) during Q_2_ and Q_3_.

			**INI**	**BL1**	**BL2**	**BL3**	**BL4**	**BL5**	**POST**
FRES (N)	R	Q_2_	23.12 (6.77)^12345^	**26.57 (8.35)** ^**345**^	**34.00 (16.16)** ^**45**^	**36.81 (11.90)** ^**45**^	**42.50 (13.73)**	**43.39 (16.80)**	23.28 (7.84)
		Q_3_	31.36 (7.99)^12345^	**38.29 (9.63)** ^**2345**^	**50.04 (16.39)** ^**45**^	**53.90 (17.92)** ^**45**^	**60.58 (17.27)**	**60.35 (16.78)**	32.32 (8.95)
	L	Q_2_	22.15 (5.70)^12345^	**26.81 (8.01)** ^**2345**^	**32.80 (13.81)** ^**45**^	**35.07 (12.37)** ^**5**^	**40.65 (13.26)**	**43.26 (16.36)**	22.94 (6.91)
		Q_3_	29.38 (6.05)^12345^	**36.77 (8.95)** ^**2345**^	**46.49 (13.50)** ^**45**^	**50.99 (16.75)** ^**45**^	**57.33 (17.30)**	**59.88 (16.45)**	31.00 (7.98)
FTAN (N)	R	Q_2_	41.62 (7.16)^12345^	**46.50 (9.82)** ^**2345**^	**54.61 (13.88)** ^**45**^	**57.18 (15.00)** ^**45**^	**63.25 (16.21)**	**62.99 (15.82)**	42.53 (8.08)
		Q_3_	44.52 (10.70)^12345^	**53.50 (13.88)** ^**2345**^	**66.31 (20.32)** ^**45**^	**70.63 (25.00)** ^**5**^	**77.53 (23.71)**	**77.84 (19.82)**	46.31 (12.72)
	L	Q_2_	38.52 (7.33)^12345^	**44.78 (8.55)** ^**2345**^	**52.38 (11.87)** ^**5**^	**54.34 (14.35)** ^**5**^	**59.18 (15.33)**	**61.12 (15.38)**	40.27 (7.18)
		Q_3_	41.66 (9.67)^12345^	**51.26 (14.33)** ^**2345**^	**63.41 (20.03)** ^**45**^	**68.30 (26.09)** ^**5**^	**74.11 (24.59)** ^**5**^	**78.04 (21.99)**	44.56 (13.25)
MEF	R	Q_2_	0.33 (0.13)^345^	**0.34 (0.12)** ^**345**^	**0.39 (0.19)**	**0.43 (0.14)**	**0.46 (0.15)**	**0.48 (0.18)**	0.32 (0.15)
		Q_3_	0.52 (0.13)^345^	**0.54 (0.13)** ^**345**^	**0.59 (0.16)**	**0.61 (0.14)**	**0.63 (0.12)**	**0.61 (0.14)**	0.51 (0.15)
	L	Q_2_	0.35 (0.12)^345^	**0.37 (0.13)** ^**345**^	**0.40 (0.19)** ^**5**^	**0.43 (0.16)**	**0.48 (0.14)**	**0.50 (0.19)**	0.34 (0.14)
		Q_3_	0.53 (0.14)^345^	**0.55 (0.14)** ^**345**^	**0.57 (0.17)** ^**45**^	**0.60 (0.16)**	**0.63 (0.15)**	**0.62 (0.16)**	0.53 (0.16)

*Bold characters denote significant main effect for training intensities (p <0.05)*.

*^x^significant difference found with the training block (BLX) (p <0.0083)*.

### Shoulder Joint Moments

Average (1 SD) of each shoulder joint moment component in N.m can be found in Table 3. For the adduction/abduction moment component, significant increases during Q_3_ on the right side and Q_2_ on the left side were found between INI and BL3 and BL4. For Q_3_ on the left side, INI was significantly lower than all the training blocks. Significant differences for the internal/external rotation moment component were only found on the left side. More precisely, significant increases were found between INI and BL2–BL5 during Q_2_. During Q_3_, INI was significantly lower than all the training blocks. At the right shoulder, significant increases were found for the average flexion/extension moment component between INI and BL2–BL4 during Q_2_ and BL3 and BL5 during Q_3_. On the left side, significant augmentation between INI and all the training blocks during Q_2_ and Q_3_ were found except for BL1 during Q_2_.

**Table 3:** Average (1 SD) bilateral shoulder joint moment components in N.m during Q_2_ and Q_3_.

			**INI**	**BL1**	**BL2**	**BL3**	**BL4**	**BL5**	**POST**
ADD(+)/ABD(–)	R	Q_2_	1.32 (1.24)	1.30 (1.09)^4^	1.87 (1.76)	1.95 (1.66)	1.74 (1.40)	1.96 (1.57)	1.36 (0.98)
		Q_3_	1.94 (1.49)^34^	**2.17 (1.51)** ^**4**^	**2.53 (1.46)**	**2.84 (1.81)**	**3.24 (1.69)**	**3.16 (1.81)**	2.11 (1.33)
	L	Q_2_	1.59 (1.13)^345^	**2.23 (1.56)** ^**4**^	**2.91 (2.72)**	**3.35 (2.79)**	**3.58 (2.71)**	**3.65 (2.42)**	1.92 (1.22**)**
		Q_3_	2.84 (1.83)^12345^	**3.99 (2.34)** ^**345**^	**5.03 (2.83)** ^**45**^	**6.23 (3.97)**	**6.48 (3.39)**	**6.49 (3.77)**	3.47 (1.97)
INT (+)/EXT (–) ROTATION	R	Q_2_	4.89 (2.27)	5.01 (2.06)	5.18 (2.90)	5.87 (3.20)	6.19 (2.65)	6.01 (2.97)	4.88 (2.28)
		Q_3_	4.10 (2.12)	4.40 (1.91)	4.60 (2.35)	4.76 (2.22)	5.15 (1.92)	5.44 (2.75)	4.07 (1.94)
	L	Q_2_	7.59 (4.54)^345^	**9.36 (4.48)**	**9.88 (4.73)**	**11.93 (6.18)**	**12.79 (7.12)**	**13.03 (9.69)**	8.01 (3.04)
		Q_3_	6.35 (3.47)^12345^	**8.10 (3.44)** ^**345**^	**9.14 (4.01)** ^**45**^	**10.79 (4.45)**	**11.34 (5.21)**	**11.96 (5.10)**	6.99 (2.81)
FLEX (+)/EXT (–)	R	Q_2_	11.60 (3.92)^2345^	**12.66 (4.07)** ^**345**^	**13.89 (4.22)** ^**5**^	**15.38 (4.63)**	**16.72 (5.72)**	**16.40 (5.01)**	11.62 (3.79)
		Q_3_	8.41 (3.84)^35^	**8.98 (3.77)** ^**5**^	**10.06 (4.47)**	**10.23 (3.30)**	**10.97 (5.30)**	**12.05 (5.02)**	8.32 (3.24)
	L	Q_2_	12.84 (4.97)^12345^	**15.68 (5.08)** ^**345**^	**17.01 (5.55)** ^**45**^	**19.77 (6.65)**	**21.61 (7.58)**	**21.76 (8.99)**	13.66 (3.89)
		Q_3_	9.49 (4.49)^2345^	**11.47 (4.92)** ^**345**^	**13.00 (5.87)** ^**45**^	**14.88 (4.96)**	**15.70 (6.41)**	**16.62 (6.55)**	10.23 (4.03)

*Bold characters denote significant main effect for training intensities (p <0.05)*.

*^x^significant difference found with the training block (BLX) (p <0.0083)*.

### Shoulder Joint Forces

Table 4 shows the average (1 SD) of the shoulder joint force components in N. The anterior/posterior force component significantly increased bilaterally between INI and all the training blocks during Q_2_ and Q_3_. For the distal/proximal force component, only a significant increase between INI and BL4 was observed bilaterally during Q_3_. Significant increases between INI and BL3–BL5 were observed during Q_3_ at the right shoulder for the average medial/lateral force component. Meanwhile, at the left shoulder, significant higher average medial/lateral force components were found between INI and all the training blocks during Q_2_ and Q_3_.

**Table 4:** Average (1 SD) bilateral shoulder joint force components in N.m during Q_2_ and Q_3_.

			**INI**	**BL1**	**BL2**	**BL3**	**BL4**	**BL5**	**POST**
ANT(+)/POST(–)	R	Q_2_	26.21 (8.07)^12345^	**31.08 (8.58)** ^**2345**^	**37.69 (10.42)** ^**45**^	**42.09 (12.95)** ^**4**^	**47.48 (13.57)**	**44.91 (12.35)**	27.59 (8.69)
		Q_3_	27.53 (8.90)^12345^	**34.38 (10.83)** ^**2345**^	**42.09 (16.88)** ^**45**^	**45.56 (17.70)** ^**5**^	**49.44 (16.39)**	**51.55 (14.29)**	29.10 (11.27)
	L	Q_2_	24.13 (9.70)^12345^	**29.54 (9.78)** ^**345**^	**34.00 (11.16)** ^**45**^	**38.55 (14.57)**	**43.00 (15.75)**	**41.84 (14.91)**	25.83 (8.98)
		Q_3_	25.06 (7.62)^12345^	**32.28 (10.40)** ^**2345**^	**39.14 (15.27)** ^**45**^	**43.98 (17.68)5**	**46.90 (15.44)**	**50.38 (16.98)**	27.53 (9.71)
PROX (+)/DIST (–)	R	Q_2_	26.15 (7.12)	26.18 (7.10)	21.90 (8.23)	24.25 (7.91)	24.07 (10.02)	23.60 (11.83)	26.17 (8.37)
		Q_3_	12.75 (7.48)^4^	11.85 (7.78)^24^	8.59 (6.11)	10.05 (7.52)	7.64 (5.12)	10.27 (11.68)	12.48 (9.16)
	L	Q_2_	26.45 (7.92)	27.53 (8.77)	24.83 (11.49)	26.91 (9.88)	27.08 (11.46)	25.65 (13.61)	27.23 (8.82)
		Q_3_	15.41 (8.90)^4^	**16.51 (9.70)**	**13.50 (11.64)**	**12.49 (10.35)**	**12.35 (8.05)**	**13.67 (8.19)**	15.32 (10.62)
LAT (+)/MED (–)	R	Q_2_	7.12 (3.75)	6.96 (3.14)	6.63 (3.31)	6.66 (3.15)	6.61 (3.97)	6.76 (3.53)	6.93 (3.12)
		Q_3_	−5.44 (4.73)^345^	**−7.05 (3.86)** ^**345**^	**−9.51 (5.59)**	**−10.67 (5.63)**	**−12.36 (6.86)**	**−11.36 (7.55)**	−6.66 (3.91)
	L	Q_2_	7.45 (4.66)	**6.38 (4.65)**	**6.52 (4.81)**	**7.44 (5.60)**	**7.50 (6.06)**	**7.40 (5.33)**	6.20 (5.49)
		Q_3_	−9.20 (6.15)^12345^	**−12.20 (6.88)** ^**345**^	**−16.31 (10.23)** ^**4**^	**−22.22 (17.33)**	**−23.14 (15.67)**	**−23.62 (19.76)**	−10.24 (5.53)

*Bold characters denote significant main effect for training intensities (p <0.05)*.

*^x^significant difference found with the training block (BLX) (p <0.0083)*.

## Discussion

The purpose of this study was to determine the impact of increasing force effectiveness at the pushrim by 10% during actual manual wheelchair (MWC) propulsion in experienced wheelchair users using the HB simulator on the mechanical load sustained at the shoulder. The value of the MEF obtained during INI condition compares well with previous research among individuals with a SCI where the MEF ranged from 21 to 56% (3, 5, 28). In terms of shoulder joint moments, our results are also in line with previous research that showed that the main moment components were in flexion, adduction, and internal rotation (29–31). For the shoulder net joint forces, the highest components were found in the anterior, proximal, and lateral directions that are in agreement with previous studies on individuals with a SCI (29, 30, 32).

### Haptic Biofeedback Intensity Level Influences the Mechanical Load Sustained at the Shoulder

The targeted MEF in this study was based on the earlier hypothesis postulated from a simulation study by Desroches et al. (2008) that stated that an increase of 10% in the MEF effectiveness would not yield a significant augmentation for shoulder loads. In order to reach the 10% target, the HB corresponding to 15% (BL3) had to be applied. This simulation block yielded statistically significant increases in shoulder mechanical loads. This load was found mostly in the sagittal plane (i.e., flexion moment and anterior force component). This confirms previous suggestion made in simulation and analytic studies (12–15). On average, the increases found in the moments and force ranged from 1 to 7 Nm and 5 to 11 N, respectively, which are of small amplitude and probably only have very limited effect on the risk exposure at the shoulders. Vegter et al. (33) reported a net average moment during the propulsion cycle, which varied from 12.4, 16.1, and 15.3 N.m as measured in three periods of 4 min separated by 2 min rests. These data correspond to able-bodied subjects and are slightly higher than the one presented here for our SCI subjects. Frost et al. (34) suggested that repeated tasks performed with force requirements over 10% of the maximal voluntary contraction could increase the risk of shoulder injury. The increases in moments and forces found for the BL3 training block corresponded to 9.1 and 3.8% of their respective moments and force reached during maximal voluntary propulsive moment test prior to the experiment. Thus, the advantages of an increase mechanical efficiency during propulsion outweigh the increase mechanical demand at the shoulders, as it would reduce push frequency and one could suspect that overall less work will have to be performed to cover the same distance (33). The purpose of this study was to investigate the effect of improving the MEF by using haptic feedback onto the shoulder joint moments. The authors are aware of the difficulty to fix a threshold about the joint moment during manual wheelchair propulsion and a risk of injury. It is known in ergonomic studies that risk of injury is either related to the amount of force applied, but also the repetition. In general, a task that demands 30% of maximal force at the joint is considered as fatiguing and constraining task.

The increases in the mechanical load at the shoulder found in this study, although of small amplitude, might have partly resulted in application of the external forces. Because of the nature of the HB that is to give feedback associated using force application, it is not possible to dissociate the increased force requirements in order to achieve the desired movement pattern. However, it is possible to suspect that over a longer period of time (i.e., longer training), the participants might develop the proper motor pattern that would avoid the increase resistance at the wheel and yield higher propulsion efficiency without the increase mechanical loads (35). Future studies should focus on the adaptation yielded from a longer training program that might give insight into proper future training regimen.

### Haptic Biofeedback as a Training Tool for Wheelchair Propulsion to Increase the Mechanical Efficiency

The premise behind the use of the HB is that it provides the motor system with additional proprioceptive and somatosensory cues to enhance motor planning (35). These additional cues might yield specific neural adaptations based on the desired imposed movement (36) and have a better potential for long-term residual effect when used as a training method, even more so if combined with visual feedback (10, 35, 36). These adaptations or the changes elicited when subjected to the HB might be more evident when the participants are either novice to the task or have a poor initial performance (37). As highlighted in Figure 2, a participant that was initially inefficient (i.e., poor performer; MEF = 20%) seems to benefit greatly from the HB training, whereas a participant that had initially an efficient propulsion (i.e., MEF = 50%) did not modified his response to the HB training. Thus, this suggest that the training should be adapted to each individual and future studies should focus on investigating which parameters would be more beneficial in order to optimize propulsion performance.

In this study, different blocs of haptic feedback BL1–BL5 were investigated during wheelchair propulsion. The last bloc BL5 induced a high resistance. The general idea in this study was to prove that the continuous modification of the MEF was possible, since the direction of the resultant force tended to follow the targeted direction. The targeted directions have been arbitrarily set by adding 10% to the initial MEF peak of each participant. It happens in this study that during training with BL3 block, the measured MEF was close to the arbitrarily imposed MEE target. To find out the reason of this behavior, we suggested to base this study to the general organizational principle of control. In fact, van Ingen Schenau et al. (38) have shown that many tasks have a conflicting effect in terms of orientation of reaction forces and the distribution of net joint moments either in the upper limb (push and pull) or lower limb (cycling). They have attributed a special role to biarticular muscles as responsible for the direction of the reaction forces, whereas the work done by this reaction force will be mainly realized by monoarticular muscles. It will be interesting in the future to test this hypothesis with either muscular activity measurement or musculoskeletal modeling approach.

In earlier study, Blouin et al. (8) have shown that some subjects keep their new MEF pattern slightly higher that the pretraining pattern [see Figure 8 in (8)] into the posteffect condition. More precisely, 7 subjects rise their MEF with respect to the initial one, whereas 11 subjects lower their MEF during the posteffect condition. It is known that learning consolidation necessitates many training periods during weeks. Unfortunately, with the data of this study, it is not possible to predict the consolidation of the new MEF pattern and future study will help to see if longitudinal training can improve the original MEF pattern.

### Limitations

This study has a few limitations. The proposed training with the HB was tested on 18 participants with a SCI, which limits the generalization of this study to the other manual wheelchair users. In addition, although the parameters of the simulator were adjusted for each participant, the propulsion training on the simulator was still conducted using a single standard wheelchair for all the participants. Hence, the participants may have been less adapted to propelling a wheelchair that was not theirs. Future studies should focus on the adaptation yielded from a longer training program that might give insight into proper future training regimen. Moreover, in the inverse dynamic model, we do not consider all the shoulder girdle joints and possible contribution of clavicle and scapula motions to glenohumeral loading.

## Conclusion

Increases in shoulder loads were found for the corresponding training blocks but even though the percentage of the increase seems high, the amplitude of the joint moment remains under the values of wheelchair propulsion found in the literature. The use of a haptic feedback (HB) simulator is considered here as a safe approach to increase mechanical effectiveness. However, the longitudinal impact of this enhancement remains unknown for the impact on the shoulder joint. Future studies will be focused on this impact in terms of shoulder risk injury during manual wheelchair propulsion.

## Data Availability Statement

The raw data supporting the conclusions of this article will be made available by the authors, without undue reservation.

## Ethics Statement

The studies involving human participants were reviewed and approved by Ecole de technologie supérieure Centre of Interdisciplinary Research in Rehabilitation of Greater Montreal. The patients/participants provided their written informed consent to participate in this study.

## Author Contributions

RA was responsible for the collect of data, computational analysis, and writing. DG was responsible for experimental design and statistical analysis. All authors contributed to the article and approved the submitted version.

## Conflict of Interest

The authors declare that the research was conducted in the absence of any commercial or financial relationships that could be construed as a potential conflict of interest.

## Publisher's Note

All claims expressed in this article are solely those of the authors and do not necessarily represent those of their affiliated organizations, or those of the publisher, the editors and the reviewers. Any product that may be evaluated in this article, or claim that may be made by its manufacturer, is not guaranteed or endorsed by the publisher.
